# Intelligent health management technology use and depressive symptoms among older adults: the serial mediating roles of physical activity and social adaptation

**DOI:** 10.3389/fpsyg.2026.1850780

**Published:** 2026-08-10

**Authors:** Xiagang Song, Zhenjiang Tian

**Affiliations:** 1 School of Law, Shihezi University, Xinjiang, China; 2 School of Social and Public Administration, East China University of Science and Technology, Shanghai, China

**Keywords:** depressive symptoms, intelligent health management technology use, older adults, physical activity, social adaptation

## Abstract

**Objective:**

This study examined the association between intelligent health management technology (IHMT) use and depressive symptoms among older adults in China, focusing on the mediating roles of physical activity and social adaptation.

**Methods:**

Using data from the 2023 China Longitudinal Aging Social Survey, this cross-sectional study included 10,300 older adults aged 60 years and above. A serial mediation model was estimated using the PROCESS macro (Model 6) in SPSS 25.0. The significance of the indirect effects was tested using bootstrap methods with 5,000 resamples.

**Results:**

IHMT use was significantly associated with lower levels of depressive symptoms after adjustment for covariates. The results identified three significant indirect associations: through physical activity, through social adaptation, and sequentially through physical activity and social adaptation. IHMT use was positively associated with physical activity and social adaptation, and physical activity was positively associated with social adaptation.

**Conclusion:**

Higher levels of IHMT use were associated with fewer depressive symptoms through both independent and sequential associations involving physical activity and social adaptation. These findings should be interpreted cautiously because the cross-sectional design does not permit causal inference or establish temporal ordering. Nevertheless, the findings indicate a potentially meaningful pattern linking IHMT use, health behavior, social adaptation, and depressive symptoms among older adults.

## 1 Introduction

Population aging represents one of the most profound demographic transformations of the 21st century, with China experiencing this transition at an unprecedented pace and scale (Bai and Lei, 2020). Alongside increasing life expectancy and rapid socioeconomic change, mental health has emerged as a central component of healthy aging (Luo et al., 2021). Among mental health concerns, depressive symptoms are particularly prevalent in later life and are associated with substantial individual and societal burdens. Recent evidence suggests that nearly one in five older adults in China experiences clinically significant depressive symptoms (Tang et al., 2021), which are linked to functional decline, cognitive impairment, and increased risks of chronic disease and mortality (Guo et al., 2023; Wu et al., 2022; Zhu et al., 2024). Despite growing recognition of these challenges, scalable and contextually relevant pathways for promoting mental well-being in later life remain insufficiently understood.

The rapid development of digital health technologies has introduced new opportunities for supporting health and well-being among older adults. A substantial body of research has documented the positive role of information and communication technologies (ICT) in enhancing social connectedness and psychological well-being (Fang et al., 2018; Sims et al., 2017). However, less attention has been paid to intelligent health management technologies (IHMT), which differ from general ICT in their direct integration into everyday health monitoring and self-regulation practices. IHMT typically encompass wearable devices, biometric monitoring systems, and other sensor-based technologies that provide real-time feedback and personalized health information (Chen et al., 2022; Chen et al., 2021; Qian and Siau, 2025). By embedding health-related feedback into daily routines, IHMT use may be more directly relevant to behavioral and psychological processes associated with mental health.

Emerging evidence suggests that IHMT use may be associated with improved psychological outcomes, potentially through both behavioral and psychosocial mechanisms (Wang, 2025). From a behavioral perspective, IHMT use may encourage physical activity through self-monitoring, goal-setting, and feedback mechanisms, which have been shown to support behavior change and promote active lifestyles (Dhar et al., 2023; Ferguson et al., 2022). Physical activity, in turn, is consistently associated with lower levels of depressive symptoms through physiological, cognitive, and affective pathways (Pearce et al., 2022; Singh et al., 2023). From a psychosocial perspective, IHMT use may facilitate older adults’ adaptation to increasingly digitalized environments, enhance their sense of competence, and support social participation, thereby contributing to better social adaptation (Chen and Wang, 2024).

Importantly, behavioral and psychosocial processes are likely to be interrelated rather than independent. Participation in physical activity may provide opportunities for social participation, strengthen self-efficacy, and enhance individuals’ capacity to adapt to changing social environments (Yang et al., 2025; Zhou et al., 2024). In this sense, physical activity may be associated with social adaptation, which in turn relates to psychological well-being. However, empirical evidence examining these interrelated pathways in the context of IHMT use remains limited, particularly among older adults in China.

To address these gaps, the present study adopts a behavioral-psychosocial perspective to examine the association between IHMT use and depressive symptoms among older adults. Rather than treating technology use solely as an instrumental factor, this study conceptualizes IHMT use as an embedded form of daily health-related practice that may be associated with both behavioral activation and psychosocial adaptation. Specifically, we examine whether physical activity and social adaptation mediate the association between IHMT use and depressive symptoms both independently and serially.

Using data from the 2023 China Longitudinal Aging Social Survey (CLASS), this study aims to examine the association between IHMT use and depressive symptoms among older adults and investigate the independent and serial mediating roles of physical activity and social adaptation. By integrating behavioral and psychosocial perspectives, this study seeks to contribute to a more nuanced understanding of how IHMT use is related to mental well-being in later life.

### 1.1 The association between IHMT use and depressive symptoms

A growing body of research suggests that health-related technologies play an important role in promoting well-being in later life, including enhancing cognitive functioning and overall quality of life (Kalınkara et al., 2024; Wang et al., 2021). While prior studies have primarily focused on general information and communication technologies (ICT; Bitomsky et al., 2025), increasing attention has been directed toward intelligent health management technologies (IHMT), which are more directly embedded in everyday health practices (Anand and Krishnan, 2026). Unlike general ICT, IHMT are designed to support continuous health monitoring, feedback, and self-regulation, thereby potentially having more direct relevance to psychological functioning (Wang, 2025).

Empirical evidence suggests that IHMT use may be associated with improved psychological well-being among older adults. For example, assistive technologies such as smart mobility devices can facilitate social participation among individuals with functional limitations, which may be linked to better mental health outcomes (Chu et al., 2025). Similarly, wearable devices that provide real-time physiological feedback may enhance individuals’ awareness of their health status and contribute to a greater sense of control, which is associated with reduced uncertainty and psychological distress (Choudhury and Asan, 2021; Elgendi et al., 2026).

At the same time, the psychological implications of IHMT use are not necessarily uniformly beneficial. The reliance on continuous self-monitoring and real-time feedback requires users to interpret and respond to complex health-related information, such as physiological indicators and risk signals. In some cases, this process may lead to increased anxiety or stress, particularly when individuals misinterpret information or experience information overload (Simpson and Mazzeo, 2017). These mixed findings suggest that the association between IHMT use and depressive symptoms may depend on how individuals engage with and respond to technology-mediated health information.

Taken together, existing evidence regarding the association between IHMT use and depressive symptoms remains limited and mixed. Therefore, this study examines whether IHMT use is associated with depressive symptoms among older adults.

### 1.2 The mediating role of physical activity and social adaptation

The relationship between IHMT use and depressive symptoms is likely to involve multiple behavioral and psychosocial processes. From a behavioral activation perspective, engagement in structured and goal-directed activities is considered a key pathway for maintaining psychological well-being, particularly in later life. IHMT, characterized by real-time monitoring, personalized feedback, and motivational cues, may encourage individuals to engage in more active daily routines by facilitating self-monitoring and goal setting (Akbar et al., 2025; Guo et al., 2025). In this sense, IHMT use may be associated with higher levels of physical activity. Physical activity, in turn, has been consistently associated with lower levels of depressive symptoms through a range of mechanisms, including physiological regulation, improved mood, and reduced stress (Pearce et al., 2022; Singh et al., 2023). Accordingly, physical activity may represent an important behavioral pathway through which IHMT use is associated with depressive symptoms.

In addition to behavioral processes, IHMT use may also be linked to psychological well-being through psychosocial pathways related to social adaptation. This psychosocial mechanism can be understood through digital inclusion theory and role theory. Digital inclusion theory emphasizes that meaningful participation in a digital society involves not only access to technologies but also the development of skills, confidence, and the capacity to use digital resources in ways that support everyday life (Reuter et al., 2023; Warschauer, 2003). From this perspective, IHMT use may represent more than the adoption of health-related devices; it may also reflect older adults’ active engagement with increasingly digitalized health and social environments. By learning to use IHMT for health monitoring, feedback interpretation, and self-management, older adults may strengthen their perceived competence and reduce feelings of exclusion from digitalized social life.

Role theory provides a complementary explanation for how IHMT use may contribute to social adaptation (Biddle, 1986). Later life is often accompanied by changes in family, community, and health-related roles. When older adults are able to use IHMT to monitor their health status and participate more actively in health management, they may shift from being passive recipients of care to more active managers of their own health. This role transformation may reinforce their sense of autonomy, usefulness, and social value, thereby supporting better social adaptation. In this sense, IHMT use may help older adults maintain social connections and adapt to increasingly technology-mediated social contexts (Chen and Wang, 2024; Gao et al., 2024; Wang, 2025). These processes may be reflected in higher levels of social adaptation, which has been shown to be associated with reduced psychological distress and lower depressive symptoms (Zheng et al., 2025). Accordingly, social adaptation may represent an important psychosocial pathway through which IHMT use is associated with depressive symptoms.

### 1.3 The serial mediating role of physical activity and social adaptation

Physical activity may be positively associated with social adaptation among older adults. From the perspective of social cognitive theory, physical activity may enhance older adults’ self-efficacy, perceived control, and confidence in managing age-related challenges (Bandura, 1986). Activity theory of aging further suggests that continued engagement in meaningful activities helps older adults maintain social roles, interpersonal connections, and a sense of purpose in later life (Havighurst, 1961). In this sense, physical activity may provide psychosocial resources for social adaptation. Prior research has similarly shown that physical activity is associated not only with physical health benefits but also with opportunities for social interaction and stronger capacity to adapt to changing environments (Yang et al., 2025; Zhou et al., 2024). Together, these perspectives suggest that physical activity may support social adaptation.

Higher levels of social adaptation, in turn, may contribute to better psychological outcomes by reducing feelings of isolation, enhancing perceived social value, and supporting more effective coping with age-related challenges (Chen and Wang, 2024; Zeng and He, 2024). Taken together, these considerations offer a theoretically plausible rationale for positioning physical activity before social adaptation in the proposed serial mediation model. However, empirical evidence examining such interrelated pathways in the context of IHMT use remains limited. Therefore, this study explores whether physical activity and social adaptation play a serial mediating role in the association between IHMT use and depressive symptoms.

**Figure 1:**
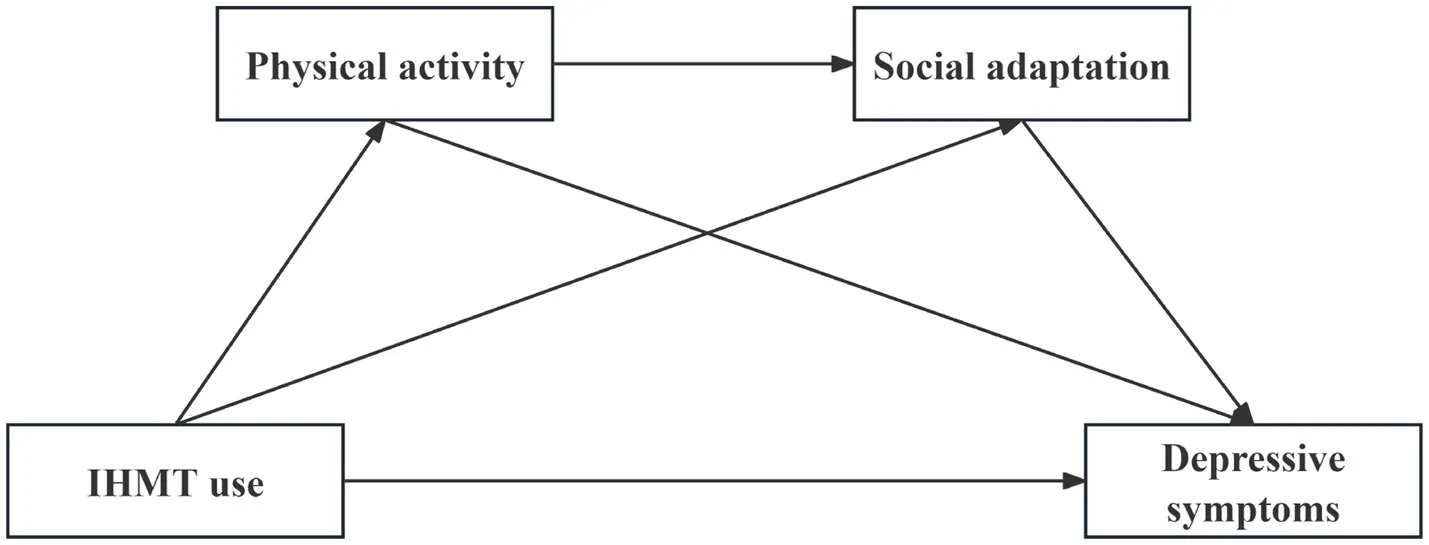
Theoretical framework of the serial mediation model.

Based on the above theoretical considerations, this study develops a conceptual framework (Figure 1) and, accordingly, proposes the following research hypotheses:

*H*1: IHMT use is negatively associated with depressive symptoms.

*H*2: Physical activity mediates the association between IHMT use and depressive symptoms.

*H*3: Social adaptation mediates the association between IHMT use and depressive symptoms.

*H*4: Physical activity and social adaptation play a serial mediating role in the relationship between IHMT use and depressive symptoms.

## 2 Materials and methods

### 2.1 Data sources

The data for this study were obtained from the 2023 wave of the China Longitudinal Aging Social Survey (CLASS), a large-scale survey of community-dwelling adults aged 60 years and older in mainland China, excluding Hainan, Xinjiang, Tibet, and the Hong Kong, Macao, and Taiwan regions. The survey was conducted by the Institute of Gerontology at Renmin University of China using a multistage stratified probability sampling design and provides extensive individual-level data for examining health and aging among older adults in China.

The 2023 CLASS initially included 11,670 respondents. First, 22 respondents younger than 60 years were excluded. Second, 350 respondents with missing data on any of the core study variables, including IHMT use, depressive symptoms, physical activity, and social adaptation, were excluded. Third, 998 respondents with missing data on any of the covariates, including gender, age, marital status, ethnicity, political affiliation, household registration, years of education, log-transformed personal income, self-rated health, number of chronic diseases, and living arrangement, were excluded. Participants with missing data on any core study variable or covariate were therefore excluded using a complete-case analysis approach. The final analytic sample comprised 10,300 participants.

### 2.2 Variable measurement

#### 2.2.1 IHMT use

Consistent with a previous study using CLASS data (Wang, 2025), IHMT use was measured through respondents’ self-reported ownership of six types of intelligent health management devices: intelligent wheelchairs, blood lipid monitors, blood pressure monitors, sleep monitors, multifunctional integrated machines, and wristbands. Because ownership of these devices is generally a prerequisite for their use, device ownership was treated as a proxy indicator of IHMT use. Each item was coded as 1 = yes and 0 = no, and the six items were summed to generate a cumulative index ranging from 0 to 6, with higher scores indicating ownership of a broader range of IHMT devices, which was interpreted as a higher level of IHMT use.

#### 2.2.2 Physical activity

Physical activity was assessed using the CLASS questionnaire item, “How often do you participate in physical activity?” Respondents reported their activity frequency on a 9-point scale: 1 = less than once a month, 2 = more than once a month but less than once a week, 3 = once a week, 4 = twice a week, 5 = three times a week, 6 = four times a week, 7 = five times a week, 8 = six times a week, and 9 = seven times or more per week. Higher scores indicate more frequent physical activity.

#### 2.2.3 Social adaptation

Social adaptation was assessed using the Social Adaptation Scale (Chen, 2008), which has been widely applied in empirical studies in the Chinese context (Bai, 2025; Yu et al., 2025; Zheng et al., 2025). The scale comprises eight items capturing different dimensions of social adaptation, including willingness to participate in community activities, desire to contribute to society, learning enthusiasm, perceived social value, adaptation to social changes, openness to new ideas, responsiveness to social policies, and perceptions of age-friendly social changes. Each item was rated on a 5-point Likert scale ranging from 1 = totally disagree to 5 = totally agree. The first four items were positively worded (e.g., “I am willing to engage in certain work in the village/community if given the opportunity”), whereas the remaining four items were negatively worded (e.g., “The growing number of new social policies makes it harder for me to adjust”) and were reverse-coded. A composite score was calculated by averaging the eight items, resulting in a score ranging from 1 to 5, with higher scores indicating greater social adaptation among older adults. In the present study, the scale demonstrated acceptable internal consistency, with a Cronbach’s alpha of 0.76.

#### 2.2.4 Depressive symptoms

Depressive symptoms were assessed using the Center for Epidemiologic Studies Depression Scale (CES-D), which has been widely validated among older adults in China (Zhu et al., 2025). The scale comprises nine items capturing a range of emotional and physiological symptoms associated with depressive symptoms, including six negative affective and somatic symptoms and three positive affective states. Each item is rated on a 3-point scale: 1 (never), 2 (sometimes), and 3 (often). Items reflecting positive affect are reverse-coded. A total score is calculated by summing all nine items, resulting in a composite index ranging from 9 to 27, with higher scores indicating more severe depressive symptoms. In the present study, it demonstrated acceptable internal consistency (Cronbach’s alpha = 0.70).

#### 2.2.5 Control variables

Drawing on previous studies examining the association between digital health technology use and health outcomes among older adults (Wang, 2025), this study included demographic characteristics, socioeconomic status, health-related factors, and social conditions as control variables. These variables may be associated with both older adults’ access to and use of IHMT and their depressive symptoms, and were therefore adjusted for in the analysis. Demographic characteristics included gender (male = 1, female = 0), age (in years), marital status (married/cohabiting = 1, other = 0), ethnic minority status (minority = 1, Han = 0), Communist Party membership (member = 1, non-member = 0), and household registration type (urban = 1, rural = 0). Socioeconomic status was measured using years of formal education and annual personal income. Annual personal income was log-transformed prior to analysis to reduce right-skewness. Health-related factors included self-rated health and the number of chronic conditions. Self-rated health was measured on an ordinal scale, with higher values indicating better perceived health status. Chronic conditions were operationalized as the count of self-reported physician-diagnosed chronic diseases. Social conditions were captured by living arrangement (living alone = 1, living with others = 0). All variables’ descriptive statistics are presented in Table 1.

**Table 1:** Descriptive statistics (*N* = 10,300).

Variables	M/n	SD/%	Min	Max
Depressive symptoms	15.414	3.361	9	27
IHMT use	0.754	1.088	0	6
Physical activity	3.347	2.879	1	9
Social adaptation	3.030	0.518	1	5
Age (years)	71.442	5.992	60	101
Gender				
Female	4,935	47.913%		
Male	5,365	52.087%		
Marital status				
Other	1789	17.369%		
Married	8,511	82.631%		
Ethnicity				
Han	9,554	92.757%		
Minority	746	7.243%		
Political affiliation				
Non-CPC member	9,993	97.019%		
CPC member	307	2.981%		
Household registration (Hukou)				
Non-agricultural	4,759	46.204%		
Agricultural	5,541	53.796%		
Years of education	6.450	3.823	0	16
Log personal income	9.519	1.180	4.605	12.899
Self-rated health	3.541	0.778	1	5
Number of chronic diseases	1.845	1.442	0	14
Living arrangement				
Living with others	9,402	91.282%		
Living alone	898	8.718%		

M, Mean; SD, Standard Deviation; n, frequency; CPC, Communist Party of China.

### 2.3 Statistical analysis

All data processing and descriptive analyses were conducted using Stata 18.0. Regression models were employed to examine the association between IHMT use and depressive symptoms. To explore the potential roles of physical activity and social adaptation, mediation analyses were conducted using the PROCESS macro (Model 6). A bootstrapping procedure with 5,000 resamples was applied to estimate indirect effects and their 95% confidence intervals. The analyses did not incorporate the CLASS sampling weights, strata, or clustering variables. Therefore, the findings should be interpreted as associations observed in the analytic sample rather than as nationally representative population estimates. Given the cross-sectional nature of the data, the mediation analysis is interpreted as examining statistical indirect associations consistent with the proposed model, rather than establishing temporal ordering or causal relationships. Variance Inflation Factors (VIFs) were examined to assess multicollinearity, with values ranging from 1.03 to 1.77, indicating no serious multicollinearity concerns.

## 3 Results

### 3.1 Descriptive statistics and correlations

Table 1 presents the descriptive statistics of all the variables. A total of 10,300 respondents were included in the sample. The participants reported moderate levels of depressive symptoms, with a mean of 15.41 (SD = 3.36) and a range of 9–27. The use of IHMT was relatively low (mean = 0.75, SD = 1.09). The mean age of the sample was 71.44 years (SD = 5.99), with a range of 60 to 101 years. Among the participants, 52.09% were male and 82.63% were married. In terms of social and health characteristics, the respondents reported moderate levels of physical activity (mean = 3.35, SD = 2.88) and social adaptation (mean = 3.03, SD = 0.52). The vast majority of the sample (92.76%) belonged to the Han ethnicity, and 2.98% were members of the Communist Party of China (CPC). Regarding household registration, 53.80% held agricultural Hukou. The average duration of education was 6.45 years (SD = 3.82). The mean log of individual annual income was 9.52 (SD = 1.18). Additionally, the mean self-rated health score was 3.54 (SD = 0.78), and the average number of chronic diseases was 1.85 (SD = 1.44). Finally, most participants (91.28%) lived with others, while 8.72% lived alone.

Table 2 presents the results of the correlation analysis for key variables. Among the sampled older adults, IHMT use was significantly correlated with lower levels of depressive symptoms (*r* = −0.200, *p* < 0.001). Similarly, both physical activity (*r* = −0.099, *p* < 0.001) and social adaptation (*r* = −0.184, p < 0.001) were negatively correlated with depressive symptoms. In terms of positive associations, IHMT use was positively correlated with physical activity (*r* = 0.117, *p* < 0.001) and social adaptation (*r* = 0.142, *p* < 0.001). Furthermore, a significant positive correlation was observed between physical activity and social adaptation (*r* = 0.087, *p* < 0.001). These correlations provide preliminary support for our proposed hypotheses, laying the groundwork for the subsequent path analysis.

**Table 2:** Correlation analysis of key variables.

Variables	Depressive symptoms	IHMT use	Physical activity	Social adaptation
Depressive symptoms	1			
IHMT use	−0.200^***^	1		
Physical activity	−0.099^***^	0.117^***^	1	
Social adaptation	−0.184^***^	0.142^***^	0.087^***^	1

*p < 0.05, **p < 0.01, ***p < 0.001.

### 3.2 Regression analysis of the mediation model

**Figure 2:**
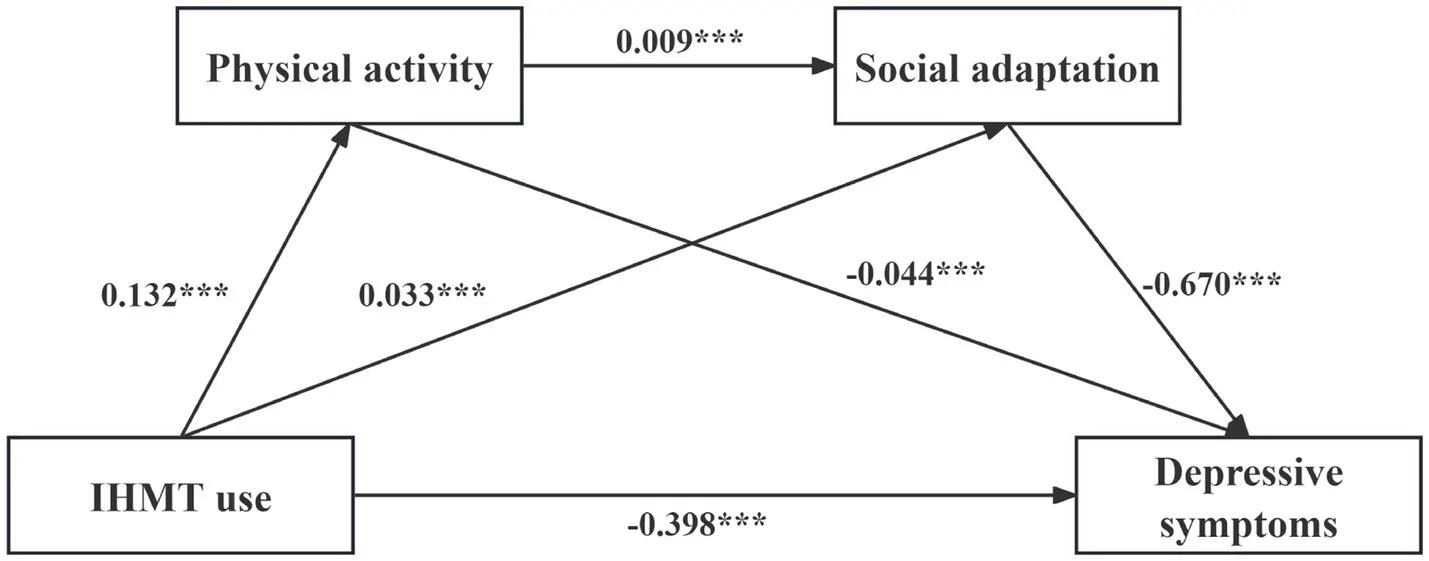
Serial mediation model of IHMT use and depressive symptoms. *p < 0.05, ** *p* < 0.01, *** *p* < 0.001.

Table 3 displays the results of multiple linear regression analyses. In Model 1, IHMT use was significantly and negatively associated with depressive symptoms (*B* = −0.426, *p* < 0.001), supporting H1. Model 1, which included IHMT use and all covariates, accounted for 14.0% of the variance in depressive symptoms. Model 2 and Model 3 showed that IHMT use was significantly and positively associated with physical activity (*B* = 0.132, *p* < 0.001) and social adaptation (*B* = 0.033, *p* < 0.001), respectively. In Model 4, after physical activity and social adaptation were included, the association between IHMT use and depressive symptoms remained significant (*B* = −0.398, *p* < 0.001). Both physical activity (*B* = −0.044, *p* < 0.001) and social adaptation (*B* = −0.670, *p* < 0.001) were significantly and negatively associated with depressive symptoms. Among the variables, age, marital status, education, and self-rated health were also significantly associated with depressive symptoms across the models. These associations are illustrated in Figure 2.

**Table 3:** Multiple linear regression models for depressive symptoms, physical activity, and social adaptation.

Variables	Model 1 (Depressive symptoms)	Model 2 (Physical activity)	Model 3 (Social adaptation)	Model 4 (Depressive symptoms)
IHMT use	−0.426^***^ (0.031)	0.132^***^ (0.028)	0.033^***^ (0.005)	−0.398^***^ (0.031)
Physical activity			0.009^***^ (0.002)	−0.044^***^ (0.011)
Social adaptation				−0.670^***^ (0.061)
Age	0.041^***^ (0.006)	−0.027^***^ (0.005)	−0.003^***^ (0.001)	0.038^***^ (0.006)
Gender	−0.119 (0.063)	−0.066 (0.056)	−0.005 (0.010)	−0.125^*^ (0.063)
Marital status	−0.321^**^ (0.108)	0.210^*^ (0.096)	−0.011 (0.017)	−0.319^**^ (0.107)
Ethnicity	0.810^***^ (0.120)	−0.884^***^ (0.107)	0.023 (0.019)	0.782^***^ (0.119)
Political affiliation	−0.059 (0.185)	0.921^***^ (0.165)	0.081^**^ (0.030)	0.041 (0.184)
Household registration	0.084 (0.071)	−0.873^***^ (0.063)	−0.007 (0.011)	0.036 (0.071)
Years of education	−0.058^***^ (0.010)	0.040^***^ (0.009)	0.012^***^ (0.002)	−0.047^***^ (0.010)
Log personal income	−0.217^***^ (0.029)	−0.107^***^ (0.026)	0.025^***^ (0.005)	−0.205^***^ (0.028)
Self-rated health	−0.896^***^ (0.042)	0.100^**^ (0.037)	0.098^***^ (0.007)	−0.826^***^ (0.042)
Number of chronic diseases	0.110^***^ (0.023)	−0.018 (0.020)	0.026^***^ (0.004)	0.127^***^ (0.023)
Living arrangement	0.382^**^ (0.139)	0.049 (0.124)	−0.025 (0.022)	0.368^**^ (0.138)
Constant	18.371^***^ (0.581)	5.990^***^ (0.520)	2.518^***^ (0.094)	20.354^***^ (0.601)
*N*	10,300	10,300	10,300	10,300
*R* ^2^	0.140	0.062	0.072	0.151

Unstandardized regression coefficients (B) are reported, with standard errors in parentheses; ^*^ *p* < 0.05, ^**^ *p* < 0.01, ^***^ *p* < 0.001.

### 3.3 Testing the significance of mediation effects

The mediation effect was tested using the PROCESS macro (Model 6) for SPSS (Hayes, 2017). A bootstrap method with 5,000 resamples was employed to estimate the 95% confidence intervals (CI) for the indirect effects. As shown in Table 4, the total indirect effect of IHMT use on depressive symptoms was −0.028 (95% CI [−0.037, −0.020]), accounting for 6.64% of the total effect.

**Table 4:** Bootstrap analysis for testing the significance of mediation effects.

Path	Effect size	SE/Boot SE	95% CI		Relative effect
			Lower	Upper	
Total effect	−0.4263	0.0309	−0.4869	−0.3658	100%
Direct effect	−0.3980	0.0308	−0.4584	−0.3377	93.36%
Total indirect effect	−0.0283	0.0044	−0.0374	−0.0201	6.64%
IHMT use → PA → DEP	−0.0058	0.002	−0.0101	−0.0023	1.36%
IHMT use → SA → DEP	−0.0218	0.0038	−0.0297	−0.0147	5.11%
IHMT use → PA → SA → DEP	−0.0008	0.0003	−0.0014	−0.0003	0.19%

PA, Physical activity; SA, Social adaptation; DEP, Depressive symptoms.

Three significant indirect pathways were identified. First, IHMT use was associated with depressive symptoms through physical activity (indirect effect = −0.006, 95% CI [−0.010, −0.002]), supporting H2. Second, a significant indirect association was observed through social adaptation (indirect effect = −0.022, 95% CI [−0.030, −0.015]), supporting H3. Third, a sequential mediation pathway via physical activity and social adaptation was also significant (indirect effect = −0.001, 95% CI [−0.001, −0.000]), supporting H4. The indirect association through social adaptation was the largest, accounting for 5.11% of the total effect.

## 4 Discussion

Using data from the 2023 CLASS, this study examined the association between IHMT use and depressive symptoms among older adults in China and the mediating roles of physical activity and social adaptation. The results showed three main findings. First, IHMT use was significantly associated with lower levels of depressive symptoms among older adults. Second, significant indirect associations were observed separately through physical activity and social adaptation. Third, the results identified a theoretically informed serial association in which physical activity preceded social adaptation. Taken together, these findings support the proposed behavioral-psychosocial integration perspective and suggest that IHMT use may be associated with mental well-being through interconnected behavioral and psychosocial processes. Given the cross-sectional design, however, the proposed sequence should be interpreted as a theoretically informed ordering rather than a confirmed temporal process.

### 4.1 The association between IHMT use and depressive symptoms among older adults

Our findings indicate that IHMT use is associated with lower levels of depressive symptoms among older adults in China. This pattern is broadly consistent with previous research linking health-related technologies to well-being and quality of life (Kalınkara et al., 2024). However, IHMT should be distinguished from general information and communication technologies (ICT). While ICT are often associated with well-being through social interaction and information access (Bitomsky et al., 2025), IHMT are more directly embedded in health monitoring and self-regulation practices. By providing real-time feedback on health indicators and daily behaviors, IHMT use may be associated with greater health awareness and perceived control, both of which have been linked to lower levels of psychological distress (Liu et al., 2016). In this sense, IHMT use may reflect a form of active health management associated with physiological regulation and psychological adjustment in later life.

### 4.2 The mediating role of physical activity

Physical activity mediated the association between IHMT use and depressive symptoms. The finding that IHMT use was associated with higher levels of physical activity is broadly consistent with previous research indicating that health-monitoring technologies may support behavioral self-regulation through feedback, goal setting, reminders, and self-monitoring functions (Ferguson et al., 2022; Nazaret and Sapiro, 2023). These functions may serve as technology-enabled behavioral prompts by drawing users’ attention to their health status and daily activity patterns, thereby making physical activity goals more salient and easier to monitor (Cusack et al., 2024; Dhar et al., 2023). Continuous feedback and behavioral cues may also be associated with greater awareness of sedentary behavior and stronger motivation to maintain active routines (Dhanasekaran et al., 2023; Lyons et al., 2014). Physical activity, in turn, has been consistently associated with lower levels of depressive symptoms. Several potential processes may underlie this association, including improved neurobiological functioning, more effective regulation of stress responses, enhanced self-efficacy, and greater access to psychosocial resources (Kandola et al., 2019; Schuch et al., 2018; Sun et al., 2023). From this perspective, physical activity may represent an important behavioral factor involved in the association between IHMT use and depressive symptoms.

### 4.3 The mediating role of social adaptation

Social adaptation mediated the association between IHMT use and depressive symptoms. The finding that IHMT use was associated with better social adaptation among older adults is consistent with digital inclusion theory, which emphasizes that meaningful engagement with digital technologies involves not only access but also the skills and confidence needed to participate in an increasingly digitalized society (Tirado-Morueta et al., 2026). From this perspective, IHMT use may form part of a broader pattern of adaptation to technological and social change (Félix et al., 2026; Liu et al., 2025). Learning to use and manage IHMT may be associated with greater confidence in navigating digital health information and technology-mediated social environments, which may in turn support older adults’ adjustment to age-related changes and evolving social contexts (Li et al., 2025; Shi et al., 2024; Zhang and He, 2022). The association between IHMT use and social adaptation may also be understood through the concept of relational agency (Edwards, 2005). As older adults become more familiar with IHMT for health monitoring and self-management, they may gain additional opportunities to interact with family members and healthcare providers, for example by sharing health information or discussing health-related decisions (Rising et al., 2021). From the perspective of role theory (Biddle, 1986), such engagement may be associated with a shift from being a passive recipient of care to taking a more active role in personal health management. This role-related shift may reinforce perceived competence, social belonging, and role identity. In addition, the greater functional independence associated with health-related technology use may help older adults maintain interpersonal connections and participation in everyday social life (Wang, 2025). Social adaptation may therefore represent an important psychosocial factor involved in the association between IHMT use and depressive symptoms by reducing feelings of marginalization and social withdrawal.

### 4.4 The serial mediating effect of physical activity and social adaptation

Beyond the two specific indirect associations, the analysis identified a significant serial indirect association through physical activity and social adaptation. This finding is broadly consistent with previous research suggesting that digital health technologies may have spillover associations, whereby engagement in health management is related not only to behavioral outcomes but also to broader psychological and psychosocial well-being (Kanamori et al., 2025). The association between physical activity and social adaptation may be understood through both behavioral and psychosocial processes. From a behavioral perspective, physical activity in later life is not only a health behavior but may also constitute a social practice embedded in daily life. Activities such as walking, square dancing, group exercise, and community-based fitness may create opportunities for interpersonal interaction, participation in public spaces, and engagement with community life. Through these forms of participation, older adults may be better able to maintain social roles, strengthen interpersonal ties, and remain connected to their social environment (Torres et al., 2024; Yu et al., 2025). From a psychosocial perspective, regular physical activity has been associated with better functional capacity, greater self-efficacy, and stronger confidence in managing age-related challenges, all of which may support social adaptation in later life (Özdemir et al., 2018; Zhang et al., 2025). Better social adaptation, in turn, may be associated with a stronger sense of social value and integration and lower levels of perceived exclusion and withdrawal. Taken together, these findings suggest that the association between IHMT use and depressive symptoms may involve interconnected behavioral and psychosocial processes, with physical activity representing a potential behavioral link between IHMT use and social adaptation. However, because the data were cross-sectional, the ordering of physical activity before social adaptation should be interpreted as theoretically informed rather than as a confirmed temporal sequence.

### 4.5 The magnitude of mediation effects

Notably, the mediation effects identified in this study were relatively small in magnitude. This finding should be interpreted in light of the conditions that may shape the extent to which IHMT use is associated with behavioral and psychosocial outcomes. Although IHMT use provides opportunities for health monitoring and self-management, its use may not necessarily be reflected in sustained changes in daily routines, particularly when older adults engage with these technologies only intermittently or do not integrate them into regular health management practices. Moreover, structural and technological barriers may limit the potential benefits associated with IHMT use. Older adults with limited digital literacy, lower socioeconomic resources, rural residence, or less familiarity with digital technologies may encounter difficulties in accessing, understanding, and continuously using IHMT. In addition, health-related and environmental constraints, such as chronic disease limitations, insufficient age-friendly exercise spaces, poor neighborhood walkability, and limited community-based activity programs, may further influence the extent to which IHMT use is associated with physical activity and social adaptation. Collectively, these factors may help explain why the observed indirect associations were modest in magnitude. Nevertheless, relatively small indirect effects do not necessarily diminish the theoretical or practical significance of the findings. Previous research has shown that even small effect sizes can accumulate over time, generalize across contexts, and produce meaningful impacts at the population level (Funder and Ozer, 2019; Wang et al., 2026). From a theoretical perspective, these modest indirect associations are consistent with the proposed behavioral-psychosocial integration framework, suggesting that physical activity and social adaptation may represent two pathways through which IHMT use is associated with depressive symptoms. From a practical perspective, in a country with a rapidly aging population such as China, even modest individual-level associations may translate into meaningful public health benefits when aggregated across millions of older adults.

### 4.6 Practical implications

The findings suggest that initiatives to promote IHMT use among older adults may be more effective when combined with age-friendly digital support, physical activity promotion, and programs that strengthen social adaptation. At the government and community levels, efforts should extend beyond expanding access to IHMT devices to include digital health training and community-based technical assistance, particularly for older adults with lower educational attainment, rural residence, limited digital literacy, or those living alone. Community health centers and elderly service organizations could integrate IHMT guidance with age-appropriate exercise programs, group-based activities, and peer-support services. Technology developers should simplify device interfaces, provide voice-based guidance, improve the clarity of health feedback, and offer practical recommendations that older adults can readily understand and follow. Family members may also support older adults in learning device functions, interpreting health information, and incorporating IHMT into routine health management. Collectively, these measures may help embed IHMT use within broader behavioral and psychosocial support systems rather than treating it as a stand-alone technological solution.

### 4.7 Limitations and future research

Despite the theoretical and practical implications discussed above, several limitations should be acknowledged. First, the cross-sectional design precludes causal inference and does not permit the temporal ordering among IHMT use, physical activity, social adaptation, and depressive symptoms to be empirically established. Although the serial mediation model was specified based on social cognitive theory and behavioral activation frameworks, all variables in the 2023 CLASS were measured at a single point in time. Reverse associations therefore cannot be ruled out. For example, older adults with fewer depressive symptoms may have greater motivation, cognitive resources, and perceived control, making them more likely to use IHMT, engage in physical activity, and adapt to social changes. In addition, unobserved factors such as digital literacy, health consciousness, family support, cognitive function, and access to community resources may be associated with both IHMT use and depressive symptoms. Although the analyses adjusted for a range of demographic, socioeconomic, health-related, and social covariates, residual confounding may remain. Accordingly, the mediation findings should be interpreted as statistical indirect associations consistent with the theoretically specified model rather than as evidence of temporal sequencing or causal mechanisms. Future research should use longitudinal, experimental, or intervention designs to examine the direction and temporal ordering of these associations more rigorously.

Second, the study relied primarily on self-reported measures, which may be subject to recall bias, reporting error, and social desirability bias. Respondents may overreport socially desirable behaviors, such as physical activity and positive social adaptation, while underreporting depressive symptoms because of stigma surrounding mental health in later life. Self-reported IHMT use may also depend on respondents’ understanding and recognition of different intelligent health management technologies and services. Such measurement bias could either attenuate or inflate the observed associations. For example, respondents who are more physically active or socially adaptive may be more likely to report both IHMT use and favorable mental health, potentially producing upwardly biased estimates. Conversely, difficulty recalling or accurately identifying technology use may weaken the estimated association between IHMT use and depressive symptoms. Future studies should combine self-reported information with objective or externally validated measures, such as device-recorded usage logs, wearable-based physical activity data, clinical assessments of depressive symptoms, and validated measures of digital literacy.

Third, the measurement of IHMT use has some limitations. In this study, IHMT use was operationalized as a cumulative index based on respondents’ self-reported ownership of six types of intelligent devices related to health management and eldercare. Consistent with previous studies using device-based indicators to measure smart eldercare technology use (Du and Wang, 2026; Wang, 2025), device ownership was treated as a proxy for IHMT use because access is generally a prerequisite for use. However, ownership does not necessarily indicate personal or regular use, and the CLASS questionnaire does not capture the frequency, intensity, duration, or integration of device use into routine health-management practices. The findings should therefore be interpreted as associations involving the breadth of intelligent-device ownership rather than the effects of sustained or effective IHMT use. Future studies should distinguish device ownership from actual use and employ multidimensional measures of use patterns and engagement.

Fourth, the present analyses did not incorporate the CLASS sampling weights or account for stratification and clustering. Although CLASS employed a multistage stratified probability sampling design, the estimates and standard errors reported in this study were derived from unweighted analyses that did not account for the complex survey structure. Accordingly, the findings should be interpreted as associations observed within the analytic sample rather than as nationally representative population estimates. Future research should assess the robustness of these findings using survey-weighted analyses with appropriate adjustments for stratification and clustering.

## 5 Conclusion

This study found that IHMT use was associated with lower levels of depressive symptoms among older adults in China. Physical activity and social adaptation showed significant indirect associations in the association between IHMT use and depressive symptoms, both independently and serially. These findings are consistent with the proposed behavioral-psychosocial integration perspective, suggesting that IHMT use may be related to mental well-being through interconnected behavioral and psychosocial processes. However, given the cross-sectional design, the findings should be interpreted as statistical associations rather than evidence of temporal sequencing or causal mechanisms. From a practical perspective, efforts to promote IHMT use may be more beneficial when accompanied by age-friendly digital support, physical activity promotion, and programs that strengthen social adaptation among older adults.

## Data Availability

Publicly available datasets were analyzed in this study. This data can be found at: publicly available datasets were analyzed in this study. This data can be found at: http://class.ruc.edu.cn.
